# Simultaneous Determination of Multiple Active Components from Bushen Pills and Application in a Pharmacokinetic Study in Rats

**DOI:** 10.1155/2020/8882892

**Published:** 2020-07-20

**Authors:** Houli Li, Benjie Wang, Guiyan Yuan, Xiaoyan Liu, Jing Huang, Lilong Xiong, Di Zhang, Weiyi Feng, Ruichen Guo

**Affiliations:** ^1^Department of Pharmacy, The First Affiliated Hospital of Xi'an Jiaotong University, Xi'an 710061, China; ^2^Institute of Clinical Pharmacology, Qilu Hospital of Shandong University, Jinan 250012, China; ^3^Department of Pharmacy, Xi'an Central Hospita, Xi'an 710003, China; ^4^School of Chemistry, Xi'an Jiaotong University, Xi'an 710049, China

## Abstract

Bushen Pills (BSPs), as a traditional Chinese medicine (TCM), is widely used in clinic to enrich Yang, nourish Yin, stem essence, and strengthen kidneys. Two chromatographic methods, liquid chromatography-mass spectrometry (LC-MS) and liquid chromatography-tandem mass spectrometry (LC-MS/MS), were applied to analyze the multiple active components of BSPs in dosage form for quality evaluation and in rat plasma for pharmacokinetics study, respectively. Three active constituents of BSPs, including paeoniflorin (PF), berberine hydrochloride (BBR), and schizandrin (SCH), were simultaneously determined by the established LC-MS method with electrospray ionization (ESI) in positive selected ion monitoring (SIM) mode at *m*/*z* 503.1, 336.0, and 455.2. The contents of PF, BBR, and SCH were (6.112 ± 0.166) mg/g, (335.1 ± 14.95) *μ*g/g, and (5.867 ± 0.136) *μ*g/g in BSPs. On this basis, PF and BBR were selected as targeted analytes for the pharmacokinetic study of BSPs in rats. Memantine hydrochloride was used as an internal standard (IS), and the plasma samples were processed by liquid-liquid extraction with ethyl acetate. All the analytes were separated on a C_18_ reversed phase column, eluted with a mobile phase consisting of acetonitrile-formic acid (0.01%) (25 : 75, *v*/*v*), and detected by ESI in the selected ion mode with multiple reaction monitoring (MRM). The target fragment ions were *m/z* 525.3 ⟶ 449.5 for PF, 336.2 ⟶ 320.2 for BBR, and 180.1 ⟶ 163.1 for IS. The linear ranges of PF and BBR were 5–500 ng/mL and 0.1–20 ng/mL with good linearity (*r*^2^ > 0.99). No obvious matrix effect was observed, and acceptable accuracy, precision, recovery, and stability were obtained. The proposed method has been successfully applied to the pharmacokinetic study of BSPs in rats after a single dose.

## 1. Introduction

Bushen Pills (BSPs), which was modified from “Dabuyin Pills” initially recorded in *Danxi xinfa* 500 years ago, is one of the well-known traditional Chinese medicines (TCM) employed to enrich Yang, nourish Yin, stem essence, and strengthen kidneys. It is prescribed in combination with 10 medicinal crude drugs at a certain mass ratio based on the theory of traditional Chinese herbal medical science. The types and ratio of the drugs were as follows: Herba Cynomorii (9.3%), Plastrum Testudinis (9.3%), Radix Rehmanniae Praeparata (23.3%), Fructus Lycii (14.0%), Radix Asparagi (9.3%), Rhizoma Anemarrhenae (9.3%), Radix Paeoniae Alba (9.3%), Rhizoma Zingiberis (2.3%), Cortex Phellodendri Chinensis (9.3%), and Fructus Schisandrae Chinensis (4.6%) [1, 2]. The formula has the effect of ameliorating clinical symptoms such as kidney water insufficiency, Yin deficiency and Yang hyperactivity, aching and pain waist and knees, dizziness, cough, wet dream, and so on [3].

As a complex multi-TCM herbs product, BSPs contain various chemical ingredients with different core structures, causing difficulties for qualitative and quantitative analyses of components. Detecting the content of index components of BSPs could help to better control the quality of the prescription, even determine the safety and effectiveness. Moreover, studying the pharmacokinetic (PK) properties of the main bioactive constituents can be useful to explain and forecast safety, further evaluate the drug efficacy, clarify the BSPs' pharmacological mechanism, promote the clinical application, and guide rational drug usage [4]. Nowadays, mass spectrometry, as one of the most widely used analytical methods, with the further development, the identification and the quantification limit of target components has been greatly improved, no matter *in vitro* or *in vivo*.

**Figure 1:**
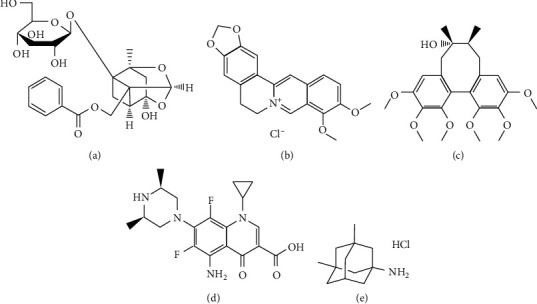
Chemical structures of PF (a), BBR (b), SCH (c), IS for LC-MS ((d), sparfloxacin), and IS for LC-MS/MS ((e), memantine hydrochloride).

According to the theory of TCM, Herba Cynomorii, Plastrum Testudinis, and Radix Rehmanniae Praeparata are the monarch herbs of BSPs. Modern studies have shown that the main pharmacological effects of Herba Cynomorii are derived from the sex hormone-like components in herbs, which can work by directly binding with sex hormone receptors, increasing the synthesis of sex hormones or improving the sensitivity of target organs to sex hormones, etc. [5]. The Plastrum Testudinis mainly contains animal glue, keratin, fat, calcium, and phosphorus, etc., which can reinforce the immune function [6]. Catalpol, rehmaionoside A & D, and verbascoside, as the main components of Radix Rehmanniae Praeparata, were decreased significantly after herb processing [7]. The active components from these three herbs are not suitable for the quantitative study. Paeoniflorin (PF, Figure 1(a)) is one main bioactive monoterpenoid glycoside in Radix Paeoniae Alba, which possesses antidepressive, anti-inflammatory, analgesic, sedative, and hypnotic, immunomodulatory activities [8–10]. Berberine hydrochloride (BBR, Figure 1(b)) is one of the most abundant and bioactive alkaloids in Cortex Phellodendri Chinensis, which is proved to show antidiabetic, antibacterial, and antihypertensive activities [11–13]. Schizandrin (SCH, Figure 1(c)), which exhibited antioxidization, enhancing immunity, and antitumor effects, is a major bioactive lignan in Fructus Schisandrae Chinensis [14, 15]. Up to now, some methods based on LC-MS or LC-MS/MS have been previously described to quantify PF, BBR, and SCH in the preparations or biological samples in other TCM formula or single herbs [16–20]. However, there is still no report on determining the three active ingredients simultaneously for content determination or pharmacokinetic investigations.

In this study, we established a direct and effective LC-MS method to quantify three major ingredients of BSPs prescription simultaneously. Subsequently, PF and BBR were selected as the index analytes in biological samples, and another simple, reliable, and rapid analytical method using LC-MS/MS technology for determination of PF and BBR in rat plasma was developed and fully validated. The proposed method was then used to investigate the PK properties of the two components after the oral administration of BSPs in rats. It is expected that the results of this study will provide valuable information on the quality control of the prescription and further facilitate the apprehension of the pharmacological mechanism and clinical application of BSPs.

## 2. Materials and Methods

### 2.1. Chemicals, Reagents, and Animals

Reference compounds of paeoniflorin (PF, Lot No. 110736–200732), berberine hydrochloride (BBR, Lot No. 110713–200609), and schizandrin (SCH, Lot No. 110857–200608) were purchased from the National Institute for Food and Drug Control (Beijing, China). Sparfloxacin (98.6%, Internal standard, IS, LC-MS) and memantine hydrochloride (99.8%, Internal standard, IS, LC-MS/MS) were provided by Dandong Yichuang Pharmaceutical Co., Ltd and Shanghai Fudan Forward Pharmaceutical Co., Ltd, respectively. Bushen Pills (BSPs, 0.158 g per pill) were kindly supplied by Bright Future Pharmaceutical Laboratories Ltd (Hong Kong). Methanol, acetonitrile, and ethyl acetate were HPLC grade from J. T. BAKER, USA. Formic acid, HPLC grade, was obtained from TEDIA company Inc., USA. Blank human plasma was provided by the Shandong Blood Center of China, and distilled water was purified using a Milli-Q system (Milford, USA). Other reagents were of analytical grade.

Male Wistar rats (body weight 300 ± 20 g) were obtained from the Laboratory Animal Center of Shandong University (China). The study protocol was approved by the Ethics Committee of Qilu Hospital of Shandong University, and all procedures were conducted in accordance with the Guidelines for the Care and Use of Laboratory Animals of Qilu Hosptial of Shandong University.

### 2.2. Instrumentation and Conditions

**Figure 2:**
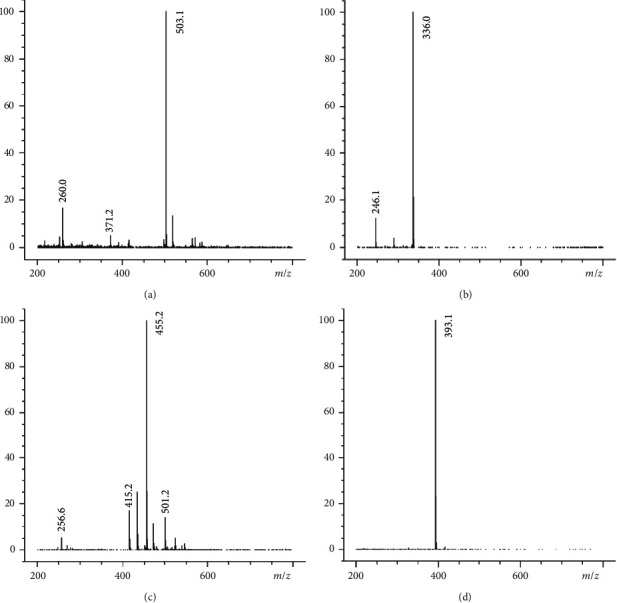
Full scan mass spectra for PF (a), BBR (b), SCH (c), and IS (d).

The LC-MS system consisted of an Agilent 1100 HPLC system and an Agilent G1946D mass spectrometer equipped with an ESI source in positive ionization mode (Agilent technologies, USA). Chromatographic separation was carried on a Waters Symmety®C_18_ column (150 mm × 3.9 mm, 5 *μ*m) and the column temperature was maintained at 25°C. The mobile phase was composed of 0.2% formic acid (A) and acetonitrile (B) with gradient elution system (0–13 min, 85%A; 13–14 min, 85% ⟶ 50%A; 14–23 min, 50%A; 23–24 min, 50% ⟶ 85%A) at a flow rate of 0.8 mL/min. For each analysis, the total run time was 24.0 min, and the injection volume was 5 *μ*L. The working parameters of the mass spectrometer detected were as follows: selected ion monitoring (SIM) mode, 50 psi of nebulizer pressure, 11 L/min of spray gas (nitrogen) flow, 350°C of spray gas temperature, 4000 V of capillary voltage, 110 V of fragmentor voltage. The protonated molecules for PF and SCH were [M+Na]^+^ with m/z at 503.1 and 455.2, and the protonated molecules for BBR and IS (sparfloxacin) were [M+H]^+^ with m/z at 336.0 and 393.1 (Figure 2). The analytical data were processed by the LC/MSD Chemstation Rev.A.10.01 software.

**Figure 3:**
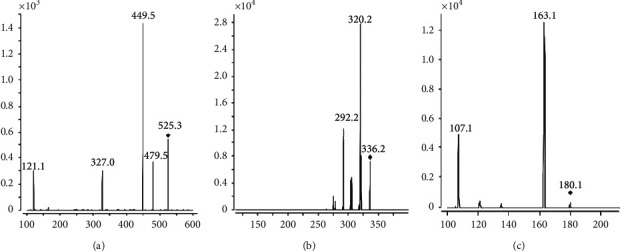
Product ion spectra of PF (a), BBR (b), and IS (c).

The LC-MS/MS system consisted of an Agilent 1200 HPLC system and an Agilent 6410 Triple Quadrupole mass spectrometer equipped with an ESI source (Agilent Technologies, USA). Chromatographic separation was carried on a Waters Symmety® C_18_ column (150 mm × 3.9 mm, 5 *μ*m) at 30°C. The mobile phase was composed of 0.01% formic acid and acetonitrile ( 75 : 25, *v*/*v*) with a flow rate of 0.5 mL/min. The injection volume was 20* μ*L, and the run time was 5.0 min. The nebulizer pressure was 50 psi, and the capillary voltage was 4000 V, with a 400 eV delta EMV. The spray gas (nitrogen) temperature was set at 350°C with a gas flow of 11 L/min. Mass spectrometric detection was operated in MRM with positive ion mode from 0 to 3.6 min for BBR and IS (memantine hydrochloride), and then with negative ion mode from 3.6 to 5.0 min for PF. The target fragment ions were *m/z* 525.3 ⟶ 449.5 for PF with the [M+HCOO]^−^ as the precursor ion, 336.2 ⟶ 320.2 for BBR, and 180.1 ⟶ 163.1 for IS (Figure 3) with the [M+H]^+^ as the precursor ion. The fragmentor and collision energy of PF, BBR, and IS were 110, 117, 120 V, and 28, 8, 18 eV, respectively. The data were obtained by the Agilent 6410 Quantitative Analysis version analyst data processing software.

### 2.3. Preparation of Standard and QC Samples

A stock solution for each standard (PF, BBR, SCH, sparfloxacin, and memantine hydrochloride) was prepared at a concentration of 1.0 mg/mL in methanol. For LC-MS assay, mixed working solutions of PF, BBR, and SCH were prepared with methanol to six concentrations for construction of calibration plots in the ranges of 5.0–15.0, 0.15–0.45, and 0.06–0.18 *μ*g/mL. The IS solution of sparfloxacin was diluted with methanol to yield a concentration of 1.0 *μ*g/mL. For LC-MS/MS assay, mixed working solutions containing 0.05–5.0 *μ*g/mL PF and 1–200 ng/mL BBR were also obtained with methanol. The IS working solution of memantine hydrochloride was diluted with the mobile phase to a concentration of 100 ng/mL. 20 *μ*L of each mixed working solution dried with nitrogen gas and 200 *μ*L blank plasma was added to get final concentration of calibration samples containing PF 5, 10, 20, 50, 100, 200, 500 ng/mL and BBR 0.1, 0.5, 1.0, 2.0, 5.0, 10.0, 20.0 ng/mL. The low, middle, and high quality control (QC) samples containing PF 6, 40, and 400 ng/mL and BBR 0.2, 4.0, and 16.0 ng/mL were prepared independently in the same fashion. All stock and working solutions were stored at 4°C in the refrigerator.

### 2.4. Application of the LC-MS Method for the Assay of BSPs

The fine powdered BSPs (0.75 g) was accurately weighed and transferred to a volumetric flask of 25 mL, then extracted with 20 mL of methanol-water (1 : 1, *v*/*v*) in an ultrasonic bath for 30 min, diluted with methanol-water (1 : 1, *v*/*v*) to volume and mixed. The extracted solution was centrifuged at 5000 rpm for 10 min. 40 *μ*L of the supernatant was mixed with 100 *μ*L of IS (sparfloxacin, 1.0 *μ*g/mL) and diluted to 1.0 mL with methanol-water (1 : 1, *v*/*v*) to get the final sample solution. Then 5 *μ*L of the solution was injected into the LC-MS system for analysis. Three batches of BSPs samples were selected randomly, and the contents of PF, BBR, and SCH were determined by the newly developed analytical method.

### 2.5. Application of the LC-MS/MS Method for Pharmacokinetic Study of BSPs

#### 2.5.1. Plasma Sample Preparation

Liquid-liquid extraction was used for extraction of PF, BBR, and IS from plasma. 200 *μ*L plasma samples spiked with 10 *μ*L of IS (memantine hydrochloride, 100 ng/mL) were mixed with 2.0 mL of ethyl acetate, vortexed for 3 minutes, and then centrifuged at 4000 rpm for 5 min. The supernatant (1.6 mL) was separated and blown to dryness in a water bath at 40°C with nitrogen. The residue was reconstituted with 100 *μ*L of the mobile phase, and finally, 20 *μ*L was injected into the LC-MS/MS system for analysis.

#### 2.5.2. Pharmacokinetic Study

The *in vivo* pharmacokinetic study was carried out with five male Wistar rats. They were allowed to acclimate to the housing conditions for at least 1 week and fasted for 12 h prior to the experiments with free access to water. The BSPs were ground into a powder and suspended in 0.4% carboxymethylcellulose sodium (CMC-Na) solution, then given to the rats at a dose of 5 g/kg (calculated by powder from BSPs) via intragastric gavage. This dose was equivalent to 30.55 mg/kg, 1775.55, and 29.35 *μ*g/kg PF, BBR, and SCH, respectively. 0.5 mL of blood was collected from the subclavian venous sinus at specified time intervals (0, 0.0833, 0.167, 0.333, 0.5, 0.75, 1, 1.5, 2, 3, 4, 6, 8, 12, and 24 h) after the administration of BSPs. Samples were placed into heparinized tubes and separated immediately by centrifugation (3000 rpm for 15 min). The obtained plasma samples were transferred into clean tubes and stored at −20°C for further analysis.

### 2.6. Method Validation

The LC-MS method for quantification of PF, BBR, and SCH in BSPs was validated with specificity, linearity, precision, accuracy, repeatability, and stability. The LC-MS/MS method for plasma samples was validated in compliance with the NMPA guidelines for bioanalytical method validation, including selectivity, matrix effect, linearity, recovery, precision, accuracy, and stability.

## 3. Results and Discussion

### 3.1. Target Analytes Selection

Considering the content and stability of ingredients, combined with the convenient and accessible analytical methods, the active components, including PF, BBR, and SCH, from the minister and adjuvant herbs of the formula, were selected as the target analytes for quality control and pharmacokinetics study of BSPs.

### 3.2. Optimization of the Method for BSPs Quality Evaluation

A gradient based LC-MS method was developed and validated for the determination of PF, BBR, and SCH in BSPs samples. Compared with methanol, acetonitrile could provide lower background noise and higher response for the cited analytes. Adding 0.2% formic acid to the aqueous phase could obtain the most satisfactory chromatographic peak shape and the sensitivity by enhancing the ionization of analytes. The gradient elution procedure of acetonitrile-0.2% formic acid in water was finally adopted and could successfully achieve good separation of the index components of BSPs with reasonable analysis time. Additionally, the MS conditions of the cited analytes were investigated in both positive and negative ion modes. The major *m/z* ions identified for PF, BBR, and SCH were 503.1, 336.0, and 455.2 in positive mode.

For the extraction of active components in BSPs, ultrasonication and cold immersion were compared. It was found that ultrasonication with methanol-water (1 : 1, *v*/*v*) for 30 min had better peak shape, less impurity interference, and higher recovery.

### 3.3. LC-MS Method Development

**Figure 4:**
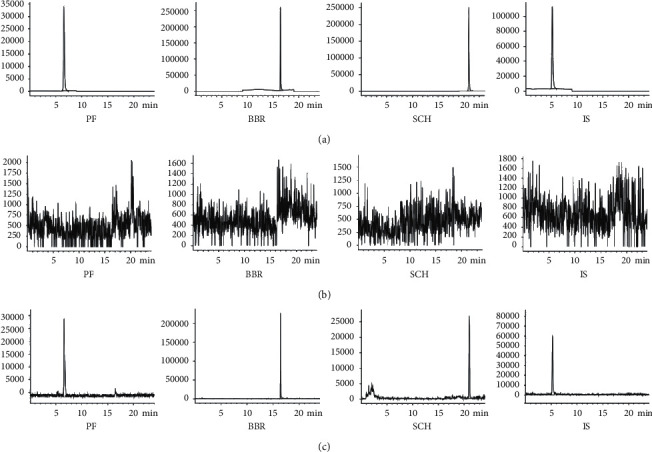
The chromatograms of related solutions for BSPs: (a) solution of mixed reference compounds, (b) blank sample solution, and (c) sample solution.

The corresponding chromatograms of mixed reference compounds solution, blank sample solution, and sample solution are shown in Figure 4. The analytes were well separated, and there are no interference peaks at the retention time of PF (6.53 min), BBR (16.41 min), SCH (20.94 min), and IS (5.14 min). The specificity of the method was adequate for analyzing these three analytes in BSPs.

The calibration curves showed excellent linearity with the concentration range of 5.0–15.0 *μ*g/mL for PF, 0.15–0.45 *μ*g/mL for BBR, and 0.06–0.18 *μ*g/mL for SCH (Table 1). The lower limits of quantification (LLOQ) for the three analytes were set at 2.5, 0.10, and 0.03 *μ*g/mL with accuracy and intra- and interday precisions within 8.5%, which was stable and sufficient for this assay.

### 3.4. Optimization of the LC-MS/MS Method

#### 3.4.1. Chromatographic and Mass Spectrometry Condition

Chromatographic and mass spectrometry conditions were also optimized for LC-MS/MS method. Acetonitrile-0.01% formic acid in water was selected to obtain a better performance in peak shape, separation and shorten analysis time. Moreover, comparing signal peak intensity in both the negative and positive ion modes, it was found that the ionization of BBR and IS was more efficient in positive ion mode, and the negative ion mode was more suitable for PF. Furthermore, the most optimal MS parameters and product ions were carefully investigated, and the extracted ion pairs of PF, BBR, and IS were chosen at *m/z* 525.3 ⟶ 449.5, 336.2 ⟶ 320.2, and 180.1 ⟶ 163.1, respectively.

#### 3.4.2. Sample Preparation

The preanalytical sample preparation for quantitative analysis by LC-MS/MS is of great importance. Protein precipitation (PPT), solid-phase extraction (SPE), and liquid-liquid extraction (LLE) were tried to get better plasma preparation. The samples pretreated for PPT with methanol and/or acetonitrile showed obvious endogenous interferences. The SPE and LLE methods were acceptable for the biosample pretreatment. However, the SPE method is extremely expensive for a large number of samples. Although the extraction recoveries for analytes were only around 40%, the LLE method with ethyl acetate was finally adopted for the sample preparation, due to its lower cost, stable extraction rate, better producibility, and lower matrix effect.

### 3.5. Method Validation for LC-MS/MS

**Figure 5:**
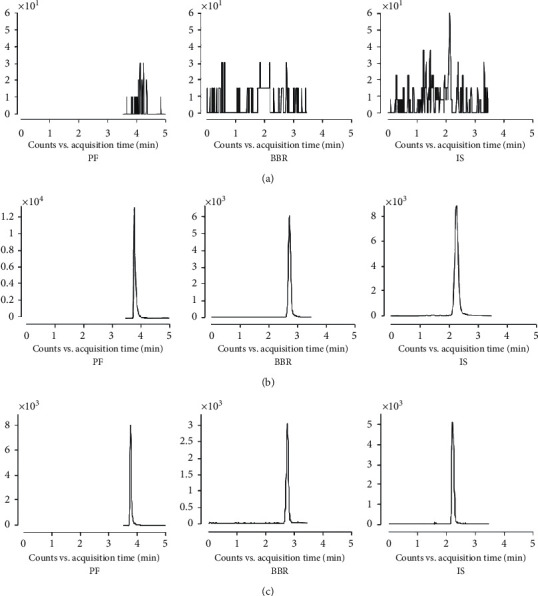
Representative multiple reaction monitoring (MRM) chromatograms of PF, BBR, and IS: (a) blank rat plasma, (b) blank rat plasma spiked with a standard solution, and (c) extracted plasma sample from a rat dosed with BSPs.

Representative MRM chromatograms of blank rat plasma, blank rat plasma spiked with PF, BBR, and IS, and extracted plasma sample from a rat dosed with BSPs are shown in Figure 5. No significant interferences were observed in the endogenous plasma substances during the analysis. Under the chromatographic conditions described, PF, BBR, and IS were eluted with the retention times of 3.8 min, 2.7 min, and 2.2 min, respectively.

The calibration curves were established, ranging from 5–500 ng/mL for PF to 0.1–20 ng/mL for BBR. Typical equations for the calibration curves were *Y* = 0.0074*X* + 0.0032 for PF, *Y* = 0.0796*X* + 9.2074 × 10^−4^ for BBR. The resulting correlation coefficients (*r*^2^) were both greater than 0.99. The LLOQ for PF and BBR was 5.0 ng/mL and 0.1 ng/mL, respectively. The matrix effect and extraction recovery were evaluated and are summarized in Table 2. The matrix effect was 96.87–107.41% for PF, 97.65–106.11% for BBR, and 94.16–100.58% for IS, thus indicating that no matrix significantly affected the drug determination in rat plasma for the present method. The extraction recoveries of PF and BBR with 3 QC concentration levels and IS ranged from 37% to 47%. Precision and accuracy were assessed on the same day or on three consecutive days and expressed by relative error (RE) and relative standard variation (RSD). The intra- and interday precision for the analytes were all less than 10.0%, and accuracies ranged from -5.01% to 3.79% (Table 2). PF and BBR were stable after storage at −20°C for 14 days, after two freeze-thaw cycles (−20°C to 25°C) and in autosampler at room temperature for 6 h after extraction. The RE for the stability of the two analytes was within ± 15%, and the RSD was less than 15.0%. These results suggested that the LC-MS/MS method described as above was satisfactory with the detection of PF and BBR in biological samples.

### 3.6. Quantitative Analysis of Active Components in BSPs

The established LC-MS method was successfully applied to quantitative analysis of the three active components. The content of PF, BBR, and SCH in three batches of commercial BSPs samples was (6.112 ± 0.166) mg/g, (335.1 ± 14.95) *μ*g/g, and (5.867 ± 0.136) *μ*g/g, respectively.

### 3.7. Pharmacokinetic Study of BSPs in Rats

**Figure 6:**
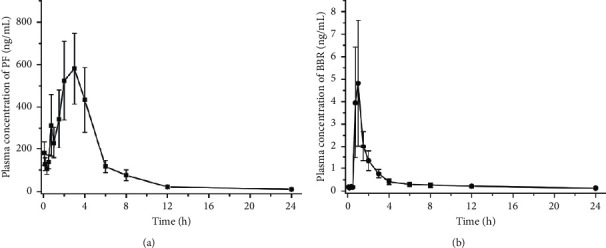
Mean plasma concentration-time curve of PF and BBR after a single dose of BSPs in rats.

The validated LC-MS/MS method was successfully applied to the pharmacokinetic study for the determination of PF and BBR concentration in rat plasma after a single oral dose of BSPs. The mean concentration-time profiles for PF and BBR are shown in Figure 6. Pharmacokinetic parameters were calculated by a standard noncompartmental model with WinNonlin Professional Version 5.2 (Pharsight Corporation, Mountain View, CA) and are summarized in Table 3.

As can be seen from Table 3, after oral administration of BSPs, the values of *T*_max_ detected in rat plasma were 2.60 h for PF and 0.90 h for BBR, indicating that BBR was absorbed more rapidly *in vivo* than PF. Besides, the values of *t*_1/2_ were 3.64 h for PF, but 9.59 h for BBR, which suggested that PF was quickly eliminated while BBR was more slowly eliminated. In addition, it showed that PF was the constituent with the greater plasma concentration and AUC than that of BBR, possibly associated with the content of PF much higher than BBR in BSPs prescription. Furthermore, a reliable LC-MS/MS method for the determination of SCH in rat plasma was also successfully established. However, the drug concentration was too low to quantify after a single dose of BSPs, resulting in the complete drug concentration-time curve not obtained. Meanwhile, the multipeak phenomenon was observed for PF. The result is similar to the PK study of PF in the previously published articles [13, 21]. Possible reasons for this phenomenon could be attributed to hepatoenteral circulation, drug-drug interaction, multiple sites absorption, etc. However, all of these probable explanations for the mechanism of multipeak phenomenon require further studies.

## 4. Conclusions

In the present study, a selective and reliable LC-MS method was developed to simultaneously detect PF, BBR, and SCH in BSPs, which is suitable for routine analysis in quality control of the pharmaceutical preparation. Another simple, rapid, and specific LC-MS/MS assay for quantification of PF and BBR in biological samples was established and fully validated, and then successfully applied to a pharmacokinetic study of BSPs after a single dose in rats. This research offers experimental evidence and valuable references for quality control and clinical use of BSPs.

## Figures and Tables

**Table 1:** The calibration curve, correlation coefficient, and linear range of three ingredients in BSPs.

Analytes	Calibration curves	Linear ranges (*μ*g/mL)	Correlation coefficient (*r*^2^)
PF	*y* = 0.01503356*x* + 0.001127	5.0–15.0	0.9975
BBR	*y* = 4.57588802*x* + 0.070837	0.15–0.45	0.9902
SCH	*y* = 1.83189923*x* + 0.0022605	0.06–0.18	0.9989

**Table 2:** Matrix effect, extraction recovery, accuracy, and precision of PF, BBR, and IS in rat plasma.

Analyte	Spiked conc. (ng/mL)	Matrix effect		Extraction recovery		Intraday (*n* = 5)		Interday (*n* = 15)	
		Mean ± SD (ng/mL)	RSD (%)	Mean ± SD (ng/mL)	RSD (%)	Accuracy (RE, %)	Precision (RSD, %)	Accuracy (RE, %)	Precision (RSD, %)
PF	6	102.23 ± 5.18	5.07	43.73 ± 1.49	3.40	−3.83	1.74	−4.87	7.03
	40	101.32 ± 4.45	4.39	45.67 ± 2.49	5.45	−3.67	2.86	−5.01	3.43
	400	101.89 ± 2.63	2.58	45.32 ± 2.25	4.97	2.71	4.08	3.44	4.16
BBR	0.2	101.88 ± 4.23	4.15	41.79 ± 2.95	7.05	−3.40	5.60	−4.50	8.83
	4.0	104.15 ± 1.57	1.51	38.47 ± 0.75	1.96	3.05	3.03	3.79	5.00
	16.0	100.21 ± 1.81	1.80	37.78 ± 0.71	1.89	−1.96	3.78	−2.27	3.82
IS	5	97.37 ± 3.21	3.30	45.00 ± 2.03	4.52	—	—	—	—

**Table 3:** The main pharmacokinetic parameters of PF and BBR after dosing with BSPs in rats (mean ± SD, *n* = 5).

Parameters	PF	BBR
*T*_max_ (h)	2.60 ± 0.55	0.90 ± 0.14
*C*_max_ (ng/mL)	692.56 ± 104.95	6.18 ± 1.89
*t*_1/2_ (h)	3.64 ± 1.87	9.59 ± 4.25
AUC_0–24_ (ng/mL·h)	2675.39 ± 465.63	9.23 ± 1.78
AUC_0–∞_ (ng/mL·h)	2732.27 ± 467.85	10.98 ± 2.45

## Data Availability

A majority of the data used to support the findings of this study are included within the article. Other data can be made available from the corresponding author upon request.
